# Supplementary material for: Degradation of Triclosan in the Water Environment by Microorganisms: A Review
Source: Microorganisms. 2022 Aug 25;10(9):1713. doi: 10.3390/microorganisms10091713 (PMC9505857; doi:10.3390/microorganisms10091713)
Supplement: Supplementary file 1 [file microorganisms-10-01713-s001.zip › microorganisms-1855807-supplementary.pdf]

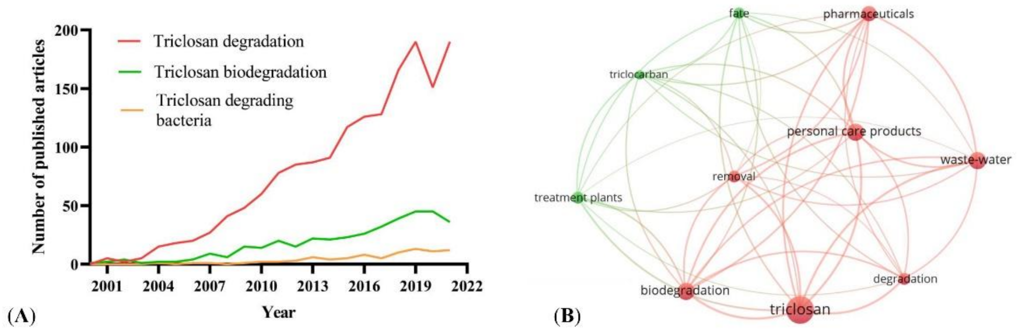

Figure S1. Number of literatures with the theme of "triclosan degradation", "triclosan biodegradation" and "triclosan degrading bacteria" in Web of Science (WOS) and co-occurrence network of top 10 keywords frequently used. (**A**) Number of literatures published during 2000-2021 with the theme of "triclosan degradation", "triclosan biodegradation" and "triclosan degrading bacteria" in WOS. (**B**) Co-occurrence network of top 10 keywords in 45 references cited in the manuscript using VOSviewer 1.6.18.0.

Table S1. The basic information of TCS.

Item	TCS
Structural formula	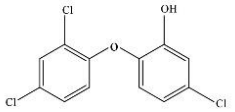
CAS	3380-34-5
Chemical formula	$C_{12}H_7Cl_3O_2$
Molecular weight	289.54
Melting point	56-60 ℃
Boiling point	344.6 ℃
Flashing point	162.2 ℃
Solubility (water)	10 mg/L
pK_a	7.9-8.1
$logK_{ow}$	4.8 at 25 °C, pH 7

Table S2. Basic information of literatures cited in the manuscript using co-occurrence network analysis.

Title	Author	Country	Journal	Published year
A novel and affordable bioaugmentation strategy with microbial extracts to accelerate the biodegradation of emerging contaminants in different media	Aguilar-Romero, I.; van Dillewijn, P.; Nesme, J.; Sorensen, S.J.; Nogales, R.; Delgado-Moreno, L.; Romero, E.	Spain, Denmark	Science of the Total Environment	2022
Family *Sphingomonadaceae* as the key executor of triclosan degradation in both nitrification and denitrification systems	Dai, H.H.; Gao, J.F.; Li, D.C.; Wang, Z.Q.; Cui, Y.C.; Zhao, Y.F.	China	Chemical Engineering Journal	2022
Triclosan: A small molecule with controversial roles	Sinicropi, M.S.; Iacopetta, D.; Ceramella, J.; Catalano, A.; Mariconda, A.; Pellegrino, M.; Saturnino, C.; Longo, P.; Aquaro, S.	Italy	Antibiotics	2022
DNA-based stable isotope probing deciphered the active denitrifying bacteria and triclosan-degrading bacteria participating in granule-based partial denitrification process under triclosan pressure	Dai, H.H.; Gao, J.F.; Li, D.C.; Wang, Z.Q.; Duan, W.J.	China	Water Research	2022
Source, bioaccumulation, degradability and toxicity of triclosan in aquatic environments: A review	Dar, O.I.; Raouf, A.; Deng, P.; Sunil, S.; Megha, A.; Kaur, A.; Jia, A.Q.; Faggio, C.	China, India, Italy	Environmental Technology & Innovation	2022
Robustness of the partial nitrification-anammox system exposing to triclosan wastewater: Stress relieved by extracellular polymeric substances and	Zhao, Y.F.; Gao, J.F.; Zhang, W.Z.; Wang, Z.Q.; Cui, Y.C.; Dai, H.H.; Li, D.C.; Zhang, Y.	China	Environmental Research	2022

resistance genes				
Biotreatment efficiency, degradation mechanism and bacterial community structure in an immobilized cell bioreactor treating triclosan-rich wastewater	Navrozidou, E.; Remmas, N.; Melidis, P.; Sylaios, G.; Ntougias, S	Greece	Environmental Technology	2021
Treatment of triclosan through enhanced microbial biodegradation	Balakrishnan, P.; Mohan, S.	India	Journal of Hazardous Materials	2021
Pharmaceuticals and personal care products' (PPCPs) impact on enriched nitrifying cultures	Lopez, C.; Nnorom, M.; Tsang, Y.F.; Knapp, C.W.	UK, China	Environmental Science and Pollution Research	2021
Triclosan weakens the nitrification process of activated sludge and increases the risk of the spread of antibiotic resistance genes	Tan, Q.Y.; Chen, J.M.; Chu, Y.F.; Liu, W.; Yang, L.L.; Ma, L.; Zhang, Y.; Qiu, D.R.; Wu, Z.B.; He, F.	China	Journal of Hazardous Materials	2021
The key active degrader, metabolic pathway and microbial ecology of triclosan biodegradation in an anoxic/oxic system	Dai, H.H.; Gao, J.F.; Wang, S.J.; Li, D.C.; Li, D.C.; Wang, Z.Q.	China	Bioresource Technology	2020
Persistence, ecological risks, and oxidoreductases-assisted biocatalytic removal of triclosan from the aquatic environment	Bilal, M.; Barcelo, D.; Iqbal, H.M.N.	China, Spain, Mexico	Science of The Total Environment	2020
Abundance of organohalide respiring bacteria and their role in dehalogenating antimicrobials in wastewater treatment plants	Zhao, S.Y.; Rogers, M.J.; He, J.Z.	Singapore	Water Research	2020
Critical review on the mechanistic photolytic and photocatalytic degradation of triclosan	Solá-Gutiérrez, C.; Schröder, S.; San-Román, M.F.; Ortiz, I.	Spain	Journal of Environmental Management	2020

Processes for the removal of triclosan in the environment and engineered systems: a review	Mulla, S.I.; Asefi, B.; Bharagava, R.N.; Saratale, G.D.; Li, J.W.; Huang, C.L.; Yu, C.P.	China, India, Republic of Korea	Environmental Reviews	2020
Removal of triclosan during wastewater treatment process and sewage sludge composting-A case study in the middle reaches of the Yellow River	Zheng, G.D.; Yu, B.; Wang, Y.W.; Ma, C.; Chen, T.B.	China	Environment International	2019
Current progress in treatment techniques of triclosan from wastewater: A review	Luo, Z.R.; He, Y.Z.; Zhi, D.; Luo, L.; Sun, Y.Q.; Khan, E.; Wang, L.; Peng, Y.T.; Zhou, Y.Y..; Tsang, D.C.W.	China, USA, UK	Science of the Total Environment	2019
Technology and principle of removing triclosan from aqueous media: A review	Quan, B.Y.; Li, X.; Zhang, H.; Zhang, C.; Ming, Y.; Huang, Y.C.; Xi, Y.N.; Xu, W.H.; Liu, Y.G.; Tang, Y.Q.	China	Chemical Engineering Journal	2019
Removal of organic micropollutants in wastewater treated by activated sludge and constructed wetlands: A comparative study	Contreras, C.R.; Lopez, D.; Leiva, A.M.; Dominguez, C.; Bayona, J.M.; Vidal, G.	Chile, Spain	Water	2019
Bioconversion of toxic micropollutant triclosan to 2,4-dichlorophenol using a wastewater isolate *Pseudomonas aeruginosa* KS2002	Kumari, R.; Sachan, S.G.	India	International Journal of Environmental Science and Technology	2019
Degradation of Triclosan from domestic wastewater by biosurfactant produced from *Bacillus licheniformis*	Jayalatha, N.A.; Devatha, C.P.	India	Molecular Biotechnology	2019
Triclosan in treated wastewater from a city wastewater treatment plant and its environmental risk assessment	Mohan, S.; Balakrishnan, P.	India	Water, Air, & Soil Pollution	2019

Isolation and identification of *Pseudomonas* from wastewater, its immobilization in cellulose biopolymer and performance in degrading triclosan	Devatha, C.P.; Pavithra, N.	India	Journal of Environmental Management	2018
Degradation of triclosan by environmental microbial consortia and by axenic cultures of microorganisms with concerns to wastewater treatment	Chen, X.J.; Zhuang, J.; Bester, K.	China, USA, Denmark	Applied Microbiology and Biotechnology	2018
Microbial degradation of triclosan by a novel strain of *Dyella* sp.	Wang, S.Z.; Yin, Y.A.; Wang, J.L.	China	Applied Microbiology and Biotechnology	2018
Emerging investigator series: dual role of organic matter in the anaerobic degradation of triclosan	Wang, L.; Xu, S.N.; Pan, B.; Yang, Y.	USA	Environmental Science: Processes & Impacts	2017
Triclosan exposure, transformation, and human health effects	Weatherly, L.M.; Gosse, J.A.	USA	Journal of Toxicology and Environmental Health, Part B	2017
Effects of different culture media on biodegradation of triclosan by *Rhodotorula mucilaginosa* and *Penicillium* sp.	Tastan, B.E.; Ozdemir, C.; Tekinay, T.	Türkiye	Water Science & Technology	2016
Characterization of triclosan metabolism in *Sphingomonas* sp. strain YL-JM2C	Mulla, S.I.; Wang, H.; Sun, Q.; Hu, A.Y.; Yu, C.P.	China	Scientific Reports	2016
Degradation of triclocarban by a triclosan-degrading *Sphingomonas* sp. strain YL-JM2C	Mulla, S.I.; Hu, A.Y.; Wang, Y.W.; Sun, Q.; Huang, S.L.; Wang, H.; Yu, C.P.	China	Chemosphere	2016

Identification of a gene cluster associated with triclosan catabolism	Kagle, J.M.; Paxson, C.; Johnstone, P.; Hay, A.G.	USA	Biodegradation	2015
Accounting for dissociation and photolysis: A review of the algal toxicity of triclosan	Roberts, J.; Price, O.R.; Bettles, N.; Rendal, C.; van Egmond, R.	UK	Environmental toxicology and Chemistry	2014
Effects of growth substrate on triclosan biodegradation potential of oxygenase-expressing bacteria	Lee, D.G.; Chu, K.H.	USA	Chemosphere	2013
Biodegradation of triclosan by a wastewater microorganism	Lee, D.G.; Zhao, F.M.; Rezenom, Y.H.; Russell, D.H.; Chu, K.H.	USA	Water Research	2012
Triclosan susceptibility and co-metabolism - A comparison for three aerobic pollutant-degrading bacteria	Kim, Y.M.; Murugesan, K.; Schmidt, S.; Bokare, V.; Jeon, J.R.; Kim, E.J.; Chang, Y.S.	Republic of Korea, South Africa	Bioresource Technology	2011
Biodegradation potential of wastewater micropollutants by ammonia-oxidizing bacteria	Roh, H.; Subramanya, N.; Zhao, F.M.; Yu, C.P.; Sandt, J.; Chu, K.H.	USA	Chemosphere	2009
Mass balance assessment of triclosan removal during conventional sewage treatment	Heidler, J.; Halden, R.U.	USA	Chemosphere	2005
Soil bacteria *Pseudomonas putida* and *Alcaligenes xylosoxidans* subsp *denitrificans* inactivate triclosan in liquid and solid substrates	Meade, M.J.; Waddell, R.L.; Callahan, T.M.	USA	FEMS Microbiology Letters	2001
Growth of a bacterial consortium on triclosan	Hay, A.G.; Dees, P.M.; Sayler, G.S.	USA	FEMS Microbiology Ecology	2001

Table S3. Information of TCS-degrading microorganisms.

Classification	Habitat	Strain	Growth substrate	Initial TCS concentration	Percentage of TCS degraded	Biodegradation rate	Degrading end products	References
G^-	Activated sludge	*Sphingopyxis* sp. KCY1	R2A + TCS	5 mg/L	100%	5 mg/L within 2 days	Cl^- and non-chlorinated, non-androgenic end products	[1][2]
G^-	Activated sludge	*Sphingomonas* sp. RD1	Complex medium containing 500 mg/L TCS	500 mg/L	/ [1]	/ [1]	CO_2	[3]
G^-	Activated sludge	*Sphingomonas* sp. PH-07	Diphenyl ether + TCS	10,000 μg/L	25%	2,500 μg/L within 8 days	4-Chlorophenol and 2,4-dichlorophenol	[4]
G^-	Activated sludge	*Sphingomonas* sp. YL-JM2C	TCS	5 mg/L	100%	5 mg/L within 72 h	CO_2	[5][6]
G^-	Activated sludge	*Citrobacter freundii* KS2003	TCS	250 mg/L	99.57±0.6%	250 mg/L within 96 h	2,4-Dichlorophenol	[7]
G^-	Activated sludge	*Pseudomonas aeruginosa* KS2002	TCS	2 g/L	99.89±0.3%	99.89±0.3% of 2 g/L TCS within 6 days	2,4-Dichlorophenol	[8]
G^-	Wastewater	*Pseudomonas* sp. TDB-5	TCS	1.5 μg/L	69%	69% of 1.5 μg/L TCS within 14 days	/ [1]	[9]

G^-	Compost	*Pseudomonas putida* TriRY	TCS	0.4 mg/L	100%	Complete removal within 6 h	/ [1]	[10]
G^-	Activated sludge	*Dyella* sp. WW1	TCS	0.2, 1.5, 5, and 10 mg/L	above 90%	/ [1]	CO_2	[11]
G^-	Activated sludge	*Providencia rettgeri* MB-IIT	TCS	10 mg/L	98%	98% removal in 24 h	Carbonxylic acid, CO_2, H_2O and Cl^-	[12]
G^-	Compost	*Alcaligenes xylosoxidans* subsp. *denitrificans* TR1	TCS	0.4 mg/L	100%	Complete removal within 24 h	/ [1]	[10]
G^-	Activated sludge	*Nitrosomonas europaea* ATCC 19178	TCS	0.5-2 mg/L	about 70%	/ [1]	/ [1]	[13]
G^-	Activated sludge	*Shewanella putrefaciens* CN32	TCS	0-15 mg/L	/ [1]	A half-life of 59 h	Dechlorinated products	[14]
G^+	Soil	*Mycobacterium vaccae* JOB5	Propane + TCS	5 mg/L	95%	0.021±0.003 mg-TCS mg-protein^{-1} d^{-1}	/ [1]	[15]
G^+	Soil	*Rhodococcus jostii* RHA1	Biphenyl + TCS	5 mg/L	64%	0.030±0.008 mg-TCS mg-protein^{-1} d^{-1}	2-Chlorohydroquinone	[15]

G+	Soil	*Paenibacillus* sp. OT2-17	TCS	2 g/L	/ [1]	Clear zone around the culture	/ [1]	[16]
G+	Wastewater	*Bacillus licheniformis* MTCC429	TCS	0.36 mg/L	100%	0.36 mg/L within 16 h	/ [1]	[17]
Fungus	Wastewater	*Rhodotorula mucilaginosa*	TCS	2.7 mg/L	48%	/ [1]	/ [1]	[18]
Fungus	Wastewater	*Penicillium* sp.	TCS	2.7 mg/L	82%	/ [1]	/ [1]	[18]

[1] '/' means that the relevant information is not mentioned.

Table S4. Basic information on Rieske type dioxygenase reference sequences.

Sequence	Group	Accession number	Organisms	References
Putative triclosan oxygenase large subunit	IA	AOT80699	*Sphingomonas* sp. RD1	[19]
Rieske 2Fe-2S domain-containing protein	IA	WP_003039765	*Sphingopyxis* sp. MC1	NCBI Refseq
Phthalate dioxygenase	IA	AAD03558	*Burkholderia cepacia*	[20]
Benzoate 1,2-dioxygenase	IB	CDY75030	*Caballeronia glathei*	NCBI Refseq
Dibenzofuran 4,4a-dioxygenase	IIA	BAJ39937	*Terrabacter* sp. DBF63	[21]
Biphenyl dioxygenase	IIB	BAA06870	*Rhodococcus jostii* RHA1	[21]
Biphenyl dioxygenase	IIB	AAB63428	*Paraburkholderia xenovorans* LB400	[23]
Naphthalene dioxygenase	III	AAS75777	*Comamonas testosteroni*	NCBI Refseq
Naphthalene dioxygenase	III	AAL07262	*Pseudomonas fluorescens*	NCBI Refseq
Naphthalene dioxygenase	III	ABA29806	*Pseudomonas* sp. LZT5	NCBI Refseq

References

[1] Zhao, F.M. Biodegradation of triclosan by a triclosan-degrading isolate and an ammonia-oxidizing bacterium. Master dissertation. Texas A&M University, **2006**.

[2] Lee, D.G.; Zhao, F.M.; Rezenom, Y.H.; Russell, D.H.; Chu K.H. Biodegradation of triclosan by a wastewater microorganism. *Water Res*. **2012**, 46, 4226-4234.

[3] Hay, A.G.; Dees, P.M.; Sayler, G.S. Growth of a bacterial consortium on triclosan. *FEMS Microbiol. Ecol*. **2001**, 36, 105-112.

[4] Kim, Y.M.; Murugesan, K.; Schmidt, S.; Bokare, V.; Jeon, J.R.; Kim, E.J.; Chang, Y.S. Triclosan susceptibility and co-metabolism - A comparison for three aerobic pollutant-degrading bacteria. *Bioresour. Technol*. **2011**, 102, 2206-2212.

[5] Mulla, S.I.; Hu, A.Y; Wang, Y.W.; Sun, Q.; Huang, S.L; Wang, H.; Yu, C.P. Degradation of triclocarban by a triclosan-degrading *Sphingomonas* sp. strain YL-JM2C. *Chemosphere* **2016**, 144, 292-296.

[6] Mulla, S.I.; Wang, H.; Sun, Q.; Hu, A.Y.; Yu, C.P. Characterization of triclosan metabolism in *Sphingomonas* sp. strain YL-JM2C. *Sci. Rep*. **2016**, 6, 21965.

[7] Kumari, R.; Sachan, S.G.; Sachan, A. Exploring triclosan degradation potential of *Citrobacter freundii* KS2003. *Int. J. Environ. Sci. Technol*. **2022**, 19, 3565-3580.

[8] Kumari, R.; Sachan, S.G. Bioconversion of toxic micropollutant triclosan to 2,4-dichlorophenol using a wastewater isolate *Pseudomonas aeruginosa* KS2002. *Int. J. Environ. Sci. Technol*. **2019**, 16, 7663-7672.

[9] Devatha, C.P.; Pavithra, N. Isolation and identification of *Pseudomonas* from wastewater, its immobilization in cellulose biopolymer and performance in degrading Triclosan. *J. Environ. Manage*. **2019**, 232, 584-591.

[10] Meade, M.J.; Waddell, R.L.; Callahan, T.M. Soil bacteria *Pseudomonas putida* and *Alcaligenes xylosoxidans* subsp. *denitrificans* inactivate triclosan in liquid and solid substrates. *FEMS Microbiol. Lett*. **2001**, 204, 45-48.

[11] Wang, S.Z; Yin, Y.A.; Wang, J. Microbial degradation of triclosan by a novel strain of *Dyella* sp. *Appl. Microbiol. Biotechnol*. **2018**, 102, 1997-2006.

[12] Balakrishnan, P.; Mohan, S. Treatment of triclosan through enhanced microbial biodegradation. *J. Hazard. Mater*. **2021**, 420, 126430.

[13] Roh, H.; Subramanya, N.; Zhao, F.M.; Yu, C.P.; Sandt, J.; Chu, K.H. Biodegradation potential of wastewater micropollutants by ammonia-oxidizing bacteria. *Chemosphere* **2009**, 77, 1084-1089.

[14] Wang, L.; Xu, S.N.; Pan, B.; Yang, Y. Emerging investigator series: dual role of organic matter in the anaerobic degradation of triclosan. *Environ. Sci. Process Impacts*. **2017**, 19, 499.

[15] Lee, D.G.; Chu, K.H. Effects of growth substrate on triclosan biodegradation potential of oxygenase-expressing bacteria. *Chemosphere* **2013**, 93, 1904-1911.

[16] Garcia, A.M.; Carlo, P.P.; Mendez, M.O. Draft genome sequence of *Paenibacillus* sp. strain OT2-17, a triclosan-degrading rhizobacterium. *Microbiol. Resour. Announc*. **2020**, 9, e01596-19.

[17] Jayalatha, N.A.; Devatha, C.P. Degradation of triclosan from domestic wastewater by biosurfactant produced from *Bacillus licheniformis*. *Mol. Biotechnol*. **2019**, 61, 674-680.

[18] Tastan, B.E.; Ozdemir, C.; Tekinay, T. Effects of different culture media on biodegradation of triclosan by *Rhodotorula mucilaginosa* and *Penicillium* sp. *Water Sci. Technol*, **2016**, 74, 473-481.

[19] Kagle, J.M.; Paxson, C.; Johnstone, P. Identification of a gene cluster associated with triclosan catabolism. *Biodegradation* **2015**, 26, 235-246.

[20] Batie, C.J.; LaHaie, E.; Ballou, D.P. Purification and characterization of phthalate oxygenase and phthalate oxygenase reductase from *Pseudomonas cepacia*. *J. Biol. Chem*. **1987**, 262: 1510-1518.

[21] Kasuga, K.; Nitta, A.; Kobayashi, M.; Habe, H.; Nojiri, H.; Yamane, H.; Omori, T.; Kojima, I. Cloning of *dfdA* genes from *Terrabacter* sp. strain DBF63 encoding dibenzofuran 4,4a-dioxygenase and heterologous expression in *Streptomyces lividans*. *Appl. Microbiol. Biotechnol*. **2013**, 97, 4485-4498.

[22] Ohmori, T.; Morita, H.; Tanaka, M.; Tomoi, M.; Miyauchi, K.; Kasai, D.; Furukawa, K.; Masai, E.; Fukuda, E. Expression in *Escherichia coli* of biphenyl 2,3-dioxygenase genes from a gram-positive polychlorinated biphenyl degrader, *Rhodococcus jostii* RHA1. *Biosci. Biotechnol. Biochem*. **2011**, 75, 26-33.

[23] Li, J.D.; Min, J.; Wang, Y.; Chen, W.W.; Kong, Y.C.; Guo, T.Y.; Mahto, J.K.; Sylvestre, M.; Hu, X.K. Engineering *Burkholderia xenovorans* LB400 BphA through site-directed mutagenesis at position 283. *Appl*. *Environ*. *Microbiol*. **2020**, 86, e01040-20.
